# Practical updates in clinical antiviral resistance testing

**DOI:** 10.1128/jcm.00728-23

**Published:** 2024-07-25

**Authors:** Hannah Wang

**Affiliations:** 1 Department of Laboratory Medicine, Cleveland Clinic, Cleveland, Ohio, USA; Vanderbilt University Medical Center, Nashville, Tennessee, USA

**Keywords:** antiviral agents, drug resistance mechanisms, human immunodeficiency virus, cytomegalovirus, herpes simplex virus

## Abstract

The laboratory diagnosis of antiviral resistance is a quickly changing field due to new drug availability, the sunsetting of older drugs, the development of novel technologies, rapid viral evolution, and the financial/logistic pressures of the clinical laboratory. This mini-review summarizes the current state of clinically available antiviral resistance testing in the United States in 2024, covering the most commonly used test methods, mechanisms, and clinical indications for herpes simplex virus, cytomegalovirus, human immunodeficiency virus, influenza, hepatitis B virus, and hepatitis C virus drug resistance testing. Common themes include the move away from phenotypic to genotypic methods for first-line clinical testing, as well as uncertainty surrounding the clinical meaningfulness of minority variant detection as next-generation sequencing methods have become more commonplace.

## INTRODUCTION

Since the discovery and development of idoxuridine in 1963 for the treatment of hepatitis B virus (HBV), over 90 antiviral drugs have been approved by the United States Food and Drug Administration (FDA) (1). These drugs constitute some of the last decade’s greatest medical achievements, giving us the ability to cure hepatitis C virus (HCV), control human immunodeficiency virus (HIV), and better prevent cytomegalovirus (CMV) disease. A central challenge in this ongoing battle against viral disease is the development of antiviral resistance. Antiviral resistance is driven by the evolutionary selection of viral mutations that confer decreased susceptibility to antiviral drugs. Testing for antiviral resistance can be used in clinical, public health, and research settings to predict treatment efficacy at the individual and population levels.

Antiviral resistance testing is dynamic, given the rapidity of viral evolution, emergence of new technologies, and changing landscape of available drugs. Clinical antiviral resistance testing is increasingly moving from phenotypic to genotypic methods and from Sanger sequencing toward next-generation sequencing (NGS) methods. These changes are controversial and come with both significant opportunities and challenges. Additionally, as new drugs are developed that have greater genetic barriers to resistance or as new viral strains take over in a population, the utility of certain antiviral resistance tests can change within a short time.

Select textbooks comprehensively discuss antiviral resistance testing for the clinical microbiologist (2, 3). This mini-review, in contrast, focuses on updates and current controversies in antiviral resistance testing methodology, as well as providing a primer on clinically available antiviral resistance testing in the United States in 2024. It includes a review of methodology; a summary of mechanisms of resistance, clinical indications, and updates and controversies in herpes simplex viruses (HSV), CMV, and HIV; an overview of the now uncommonly used antiviral resistance tests for influenza, HBV, and HCV viruses; and a discussion of future directions in clinical antiviral resistance testing.

## ANTIVIRAL RESISTANCE TESTING METHODS

### Phenotypic assays

**Fig 1:**
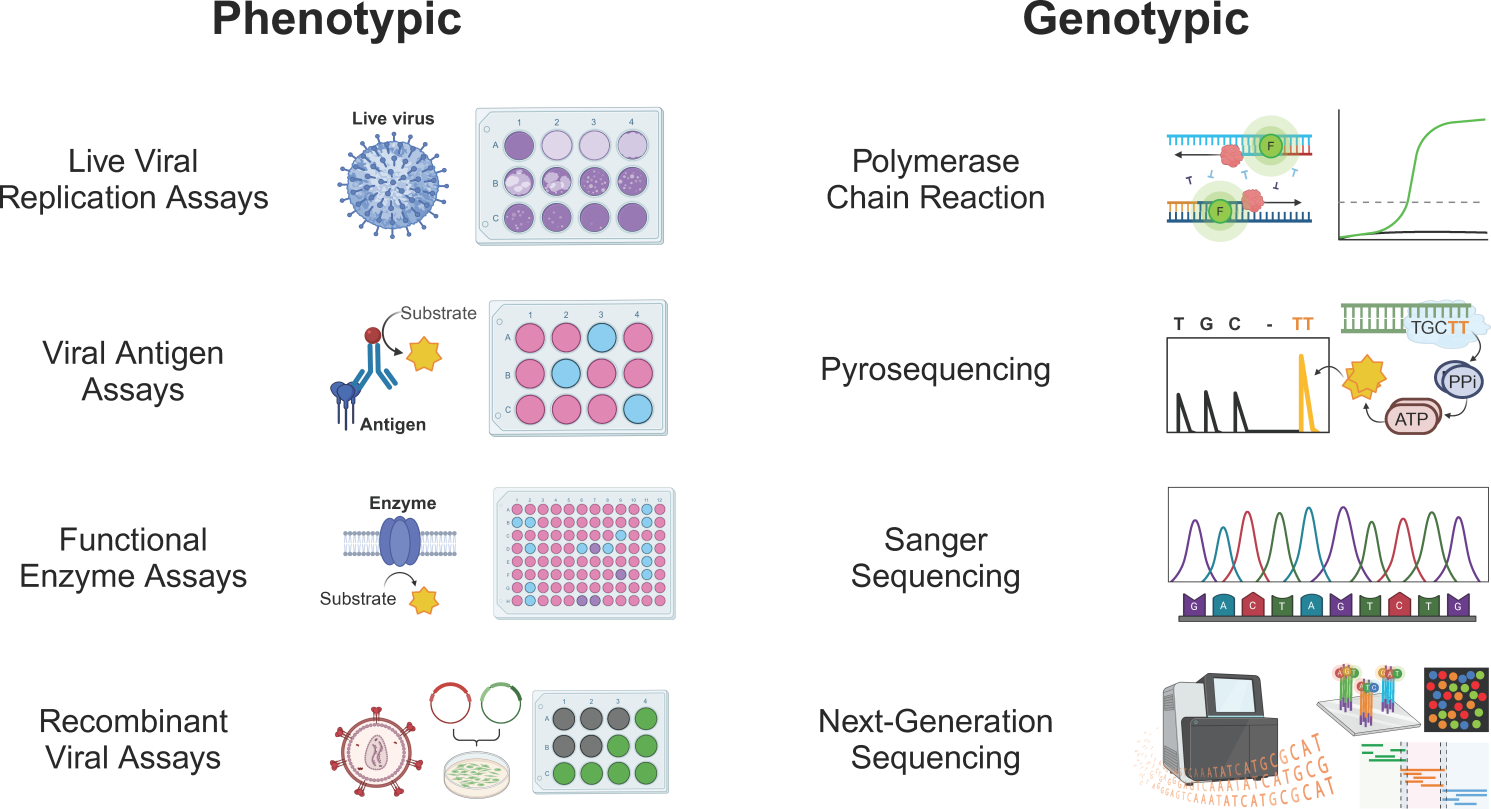
Most common phenotypic and genotypic antiviral resistance testing methods used clinically. Created with BioRender.com.

Antiviral resistance testing methods can be classified as phenotypic or genotypic (Fig. 1). The most basic phenotypic assays test for evidence of viral replication or host cell destruction (cytopathic effect), in the presence or absence of varying concentrations of drugs. An example of this is the plaque reduction assay traditionally used for HSV resistance testing. Titered virus is grown on a mammalian cell monolayer and overlaid with increasing concentrations of acyclovir or foscarnet. After incubation, plaques are counted at each drug concentration, and linear regression is performed to determine the drug concentration at which there is a 50% reduction in plaque formation compared to no-drug control wells [half-maximal inhibitory or effective concentration (IC50 or EC50)] (2, 4). Plaque counting is labor-intensive and, in many labs, has been replaced by other indirect measures of viral replication/cell death that are easier to automate, such as dye uptake, chemiluminescence, deoxyribonucleic acid (DNA) hybridization, or even quantitative polymerase chain reaction assays (5).

Other phenotypic assays that do not directly measure viral replication or cell death, but rather antigen levels or enzyme function in the presence or absence of drugs, can also be used. Enzyme immunoassays including enzyme-linked immunosorbent assays (ELISA) have been described for HSV and varicella-zoster virus (VZV) (6, 7). These assays are set up similarly to plaque reduction assays, but after incubation of virus-inoculated monolayers with varying concentrations of the drug, virus-specific antibodies and peroxidase-conjugated secondary antibodies are added to the wells, with subsequent measurement of absorbance. The quantity of viral antigen is used as a surrogate for viral replication, and an IC50 or EC50 is calculated. These ELISA-based assays demonstrated similar performance to plaque reduction assays with, in some cases, a twofold reduction in the turnaround time (7).

Another type of antigen assay used is the functional enzyme assay, which measures the enzyme activity of a virus in the presence of varying concentrations of an enzyme inhibitor. Functional enzyme assays are used for influenza neuraminidase inhibitor testing and can employ either fluorescence or chemiluminescence-based methods and report out IC50 values, which are then compared to wild-type control strains (8). The fluorescence-based assays are less costly and utilize the 4-(methylumbelliferyl)-N-acetylneuraminic acid (MUNANA) substrate, while the chemiluminescence-based methods utilize 1,2-dioxetane substrates that offer greater signal stability and wider dynamic range, but at a greater cost (8, 9).

Lastly, the most complex phenotypic methods are recombinant viral assays, where a gene segment of interest in the patient’s viral isolate is cloned into a pseudotype virus with a wild-type backbone. Live viral replication assays can then be performed with the pseudovirus, and decreased replication in the presence of drugs can be linked to mutations present in that initial isolate (2, 3). Recombinant viral assays are infrequently used in the clinical setting but play an important role in elucidating mechanisms of resistance in the research and clinical trial space.

### Genotypic assays

In general, genotypic assays provide data and yield results more quickly than phenotypic testing and have higher throughput. The simplest genotypic method involves PCR for situations where the presence of a small number of well-characterized mutations is sufficient to predict drug resistance. An example of this is the S31N mutation in the influenza A matrix gene, which mediates almost all amantadine resistance. This testing has become obsolete because this mutation became essentially ubiquitous in currently circulating influenza A strains (10).

Sanger sequencing has been classically used to identify mutations in genes of interest to predict antiviral resistance. An example of this is the ViroSeq assay used for protease and reverse-transcriptase region sequencing of the polymerase gene (*pol*) for HIV drug resistance testing. ViroSeq was FDA-approved in 2003, but the manufacturer discontinued production in 2021.

Increasingly, genotypic antiviral resistance testing is moving toward NGS methods. This type of sequencing offers the prospect of higher throughput, less manually labor-intensive bioinformatic analysis, the ability to interrogate multiple discontiguous genome regions at once, and possibly cost benefits depending on batch size. It also offers greater sensitivity to detect low-level resistant subpopulations; however, it remains unclear whether these low-level resistance mutations correlate with clinical outcomes. One additional advantage of NGS is its superior ability to resolve allele linkage between adjacent nucleotide bases in mixed populations—the inability of Sanger sequencing to do this can occasionally result in overcalling of resistance (11).

These sequencing methods are complex and, aside from a new NGS-based HIV sequencing assay from Vela Diagnostics, are not FDA-approved. Both Sanger sequencing and NGS-based methods involve the same major steps: (i) nucleic acid extraction; (ii) possible reverse transcription of RNA depending on the virus; (iii) PCR to amplify regions of interest, possibly with multiple rounds (nested PCR) to increase sensitivity; (iv) sequencing of the amplicons; (v) comparison of the patient sequences to a known wild-type reference sequence to identify mutations; (vi) determination of corresponding amino acid changes that would result from the mutations; and (vii) comparing those changes to a curated database that allows for the interpretation of resistance to specific drugs.

Antiviral resistance testing methods, both phenotypic and genotypic, tend to be centralized in large reference laboratories and academic centers and performed as lab-developed tests. As a result, the ability to act on results in a timely manner can be limited by the turnaround time. Furthermore, the testing also suffers from a lack of standardization and commutability. As these methods change over time, their respective strengths and limitations must be kept in mind as they pertain to the individual viruses discussed below.

## HERPES SIMPLEX VIRUSES

### Drugs and mechanisms of resistance

The most common drugs for the treatment of HSV-1 and HSV-2 are acyclovir (oral prodrug valacyclovir), famciclovir (oral prodrug penciclovir), cidofovir, and foscarnet (12). Acyclovir is also commonly used as a prophylactic. All these drugs ultimately inhibit viral DNA synthesis by acting upon the HSV DNA polymerase (UL30 gene). Acyclovir and famciclovir are nucleoside analogs and must first be phosphorylated by viral thymidine kinase (UL23 gene), before undergoing additional phosphorylation by cellular kinases and finally being incorporated into DNA synthesis. Once incorporated, acyclovir triphosphate halts viral DNA synthesis due to the lack of the 3′-OH group required for additional nucleotide attachment. Cidofovir is a nucleoside phosphonate analog that also terminates viral DNA synthesis but does not require viral thymidine kinase to become bioactive. Cidofovir is not FDA-approved for the treatment of HSV but is often used off-label in either intravenous (IV) or topical forms. Foscarnet is a pyrophosphate analog that also acts on HSV DNA polymerase and is FDA-approved in its IV form for the treatment of acyclovir-resistant HSV (12, 13).

Reduced susceptibility to acyclovir or penciclovir is mediated in approximately 95% of cases by mutations in UL23 (Table 1) (13, 14). The most common types of mutations conferring resistance include additions or deletions in G/C homopolymer runs, leading to premature stop codons or frameshifts; less frequently, single amino acid substitutions in conserved regions of the ATP-binding site or nucleoside-binding site are responsible. The remaining resistance is mediated by mutations in UL30, which can also confer cross-resistance to foscarnet and, rarely, cidofovir (14).

**TABLE 1:** Mechanisms of resistance for antiviral drugs with clinically available testing in the United States in 2024^*b*^

Virus	Drug/drug class	Genes (protein) mediating resistance	Most common drug resistance mutations (not comprehensive)^*a*^
HSV-1 and HSV-2	Acyclovir	*UL23* (thymidine kinase) *UL30* (DNA polymerase)	Mutations in *UL23* account for ~95% of acyclovir resistance. They include AA substitutions most commonly in ATP- and nucleoside-binding sites and insertions/deletions in G/C homopolymer regions leading to frameshifts introducing premature stop codons. Mutations in *UL30* are uncommon (~5% of acyclovir resistance) and are comprised of AA substitutions predominantly in conserved gene regions (14).
	Foscarnet	*UL30* (DNA polymerase)	Approximately half of *UL30* mutations conferring resistance to acyclovir can confer cross-resistance to foscarnet, located in conserved regions I, II, III, and VI (14).
CMV	Ganciclovir	*UL97* (protein kinase) *UL54* (DNA polymerase)	Canonical mutations M460V, H520Q, C592G, A594V, L595S, and C603W in *UL97* account for ~80% of ganciclovir resistance (15, 16). *UL54* mutations are more rare and typically seen on top of *UL97* mutations (15).
	Foscarnet and cidofovir	*UL54* (DNA polymerase)	*UL54* mutations in conserved exonuclease, palm, finger, and thumb domains, as well as amino terminal domains. Many exonuclease domain mutations confer ganciclovir–cidofovir cross-resistance (15).
	Maribavir	*UL97* (protein kinase) *UL27*	Most commonly T409M, H411Y, C480F, and F342Y in *UL97* (15, 17)
	Letermovir	*UL56, UL89, UL51* (terminase)	Most commonly, *UL56* mutations at locus C325 (15, 18)
HIV-1	NRTI and NNRTI	*pol* (reverse transcriptase)	NRTIs: M184V/I, K65R, TAMs (M41L, D67N, K70R, L210W, T215F/Y, K219Q/E), K70E, L74V/I, Y115F, T69 insertions, Q151M NNRTIs: L100I, K101E/P, K103N/S, V106A/M, Y181C/I/V, Y188L, G190A/S/E, F227L/C, M230L (19)
	PI	*pol* (protease)	V32I, M46I/L, I47V/A, G48V/M, I50L/V, I54V/L/M, L76V, V82A/F/T/S, I84V, N88D/S, L90M (19)
	INSTI	*pol* (integrase)	T66A/I/K, E92Q, G118R, E138K/A/T, G140S/A/C/R, Y143R/C/H, Q148H/R/K, N155H, R263K (19)
Influenza A and B	Neuraminidase inhibitors	*NA* (neuraminidase)	H275Y (8, 20)
	Baloxavir	*PA* (polymerase acidic protein)	I38T/M/F (10)
HBV	Nucleoside/tide analogs	*pol* (reverse transcriptase)	M204I/V, A181T/V, T184A/C/F/G/I/L/M/S, S202C/G/I, N236T, M250I/L/V, L80I/V, I169T, V173L, L180M (21–23)
HCV	NS5A inhibitor	*NS5A* (replication complex)	Y93H and other mutations in AA positions 28, 30, 31, 93 (24, 25)
	NS3/4A inhibitor	*NS3* (protease)	Q80K, R155K, and mutations in AA positions 156, 168—not associated with treatment failure in currently used drug regimens (24)
	NS5B inhibitor	*NS5B* (RNA-dependent RNA polymerase)	L159F, S282T—not associated with treatment failure in currently used drug regimens (24)

^*a*^ The mutations listed here are the most commonly reported in clinical specimens from primarily US-based studies and are not comprehensive.

^*b*^ AA, amino acid; NRTI, nucleoside/nucleotide reverse transcriptase inhibitor; NNRTI, non-nucleoside reverse transcriptase inhibitor; PI, protease inhibitor; INSTI, integrase strand transfer inhibitor.

Pritelivir, a new drug that acts on the viral helicase primase complex (UL5, UL8, and UL52 genes), was granted breakthrough therapy designation by the FDA in 2020 and is still undergoing evaluation in Phase 3 clinical trials. For patients not qualifying for clinical trials and without other treatment options, pritelivir can be obtained through an expanded access program for compassionate use. Its approval is highly anticipated because mutations in UL23 or UL30 (selected under acyclovir or foscarnet treatment) are not expected to confer cross-resistance to pritelivir (12).

Of note, although VZV is treated with similar drugs and has mechanisms of resistance similar to HSV, resistance testing is not clinically available in the United States for it currently (3).

### Clinically available testing methodologies

As of 2024 in the United States, HSV-1 and HSV-2 drug resistance testing is clinically available for acyclovir and foscarnet only (Table 2). Most testing is still performed phenotypically due to the relatively rapid growth of HSV in culture and the availability of clinical outcomes data tied to IC50 values. Still, plaque reduction and chemiluminescence assays can take up to 3 weeks to obtain a result. The evidence we have associating poor clinical response to plaque reduction assay IC50 values dates to a 1994 study of 115 HIV-infected patients with HSV disease at San Francisco General Hospital (26). Among this cohort, an acyclovir IC50 ≥2 µg/mL was associated with 95% failure to heal, whereas an acyclovir IC50 <2 µg/mL was associated with a 38% failure to heal on acyclovir therapy. Similarly, a foscarnet IC50 ≥100 µg/mL was associated with an 88% failure to heal, whereas 0% of patients with isolates with foscarnet IC50 <100 µg/mL had disease that failed to heal on foscarnet therapy (26). Phenotypic testing can be performed on isolates recovered from mucocutaneous lesional swabs or fluid, brochoalveolar lavage (BAL), or tissue.

**TABLE 2:** Phenotypic and genotypic antiviral resistance testing available clinically in the United States in 2024^*b*^

Virus	Drug/drug class	Phenotypic	Genotypic	Testing indications^*a*^
HSV-1 and HSV-2	Acyclovir	PRA or other live viral replication assays	*UL23* (thymidine kinase) sequencing (SS)	Treatment failure, immunocompromised host, keratitis (14, 27, 28)
	Foscarnet	PRA or other live viral replication assays	–	
CMV	Ganciclovir	–	*UL97* and *UL54* sequencing (SS, NGS)	Treatment failure (>1 log increase viral load or worsening end-organ disease after 2 weeks of appropriate therapy). Can also consider in cases of persistent infection/disease (29–31)
	Foscarnet and Cidofovir	–	*UL54* sequencing (SS, NGS)	
	Maribavir	–	*UL97 ± UL27* sequencing (SS, NGS)	
	Letermovir	–	*UL56* sequencing (SS, NGS)	
HIV-1	NRTI and NNRTI	Recombinant viral assay	*pol* sequencing (SS, NGS)	Acute HIV, entry to care, virologic failure, suboptimal suppression, pregnancy (32, 33)
	PI	Recombinant viral assay	*pol* sequencing (SS, NGS)	
	INSTI	Recombinant viral assay	*pol* sequencing (SS, NGS)	Virologic failure on INSTI, prior CAB-LA PrEP, suspect transmitted INSTI resistance (32, 33)
Influenza A and B	Neuraminidase inhibitors	Neuraminidase inhibition assay	*NA* sequencing (pyrosequencing, NGS)	Treatment failure, immunocompromised host (34)
	Baloxavir	Polymerase inhibition assay	*PA* sequencing (pyrosequencing, NGS)	Treatment failure, immunocompromised host (34)
HBV	Nucleoside/tide analogs	–	*pol* sequencing	Treatment failure (rare with entecavir or tenofovir) (35)
HCV	NS5A (replication complex) inhibitor	–	*NS5A* sequencing	Select situations of non-simplified treatment algorithms and/or prior to re-treatment after failure (36)
	NS3/4A (protease) inhibitor	–	*NS3* sequencing	Not routinely recommended (mutations infrequent/transient/not associated with failure) (36)
	NS5B (polymerase) inhibitor	–	*NS5B* sequencing	

^*a*^ The testing indications summarized here are based upon studies and guidelines published in the United States and may not apply to other countries where drug availability and viral strains may differ.

^*b*^ PRA, plaque reduction assay; SS, Sanger sequencing; CAB-LA PrEP, long-acting cabotegravir pre-exposure prophylaxis; *NA*, gene encoding neuraminidase; *PA*, gene encoding polymerase acidic protein.

Acyclovir genotypic resistance testing is also available in the United States for HSV-1 and HSV-2, but not for other anti-HSV drugs. The assay uses Sanger sequencing targeting the UL23 gene encoding thymidine kinase, which accounts for 95% of acyclovir resistance (14). As such, a negative result does not rule out the possibility of a UL30 mutation in DNA polymerase, which could also confer acyclovir resistance. Nevertheless, genotypic resistance testing can be useful for its quicker turnaround time compared to phenotypic methods, as well as its ability to return results for viral isolates that do not grow well in culture. The latter is not infrequent given that antiviral resistance mutations can confer a significant reduction in viral fitness (37). In addition to lesional swabs and BAL, genotypic resistance testing can be performed directly on specimen types from which HSV culture is insensitive, such as plasma/serum and cerebrospinal fluid.

HSV drug resistance is rare in immunocompetent patients. Consequently, there can be variability in methodology between labs and issues of viral heterogeneity that contribute to a lack of reproducibility in resistance testing. A European multi-center external quality assessment revealed inconsistencies between laboratories for both phenotypic and genotypic testing due to the varying ability to resolve mixed viral populations, loss of mutations conferring a drug-resistant phenotype after passaging, and errors in bioinformatic analysis (38). To minimize unnecessary variability, clinicians should consider choosing a single method or laboratory (or both) to provide testing. The threshold should be low for investigating or repeating testing that is incongruent with the clinical picture.

### Clinical indications

HSV drug resistance is primarily seen in immunocompromised patients with history of transplant or uncontrolled HIV infection, occurring in 4%–10% of patients on anti-HSV therapy. The highest risk is reported for patients with a history of allogeneic hematopoietic stem cell transplant (27). In contrast, HSV drug resistance is reported in <1% of immunocompetent patients, with the important caveat of HSV keratitis, in which acyclovir resistance can be seen in up to 6% of patients (14, 27, 28).

The prevalence of acyclovir drug resistance in immunocompetent patients has been stable at <1% since the 1980s, despite ubiquitous use for both treatment and prophylaxis. Drug resistance is almost always acquired on treatment, not transmitted, and should not be suspected in treatment-naive individuals in the absence of a strong exposure history. A recent report of possible transmission of an acyclovir-resistant HSV strain between two brothers with inherited DOCK8 immunodeficiency may represent the first reported case of transmitted drug-resistant HSV (37). In animal models, thymidine kinase-deficient viruses have been found to have impaired pathogenicity (13). This reduced fitness of drug-resistant strains likely explains the lack of transmitted drug resistance and why recurrent HSV after healing and discontinuation of antiviral therapy is often due to drug-susceptible strains (13).

Phenotypic acyclovir or foscarnet drug resistance testing, or both, should be considered in any patient whose disease does not respond to treatment, who is immunocompromised or has keratitis, and who has been on treatment or prophylaxis with these drugs and any patient with a history of drug-resistant HSV disease.

## HUMAN CYTOMEGALOVIRUS

### Drugs and mechanisms of resistance

The drugs used for CMV treatment and prophylaxis overlap with those used for HSV, with ganciclovir (oral prodrug valganciclovir) replacing acyclovir due to its greater specificity for the CMV UL97 kinase (UL97 gene). The UL97 kinase catalyzes the initial phosphorylation of ganciclovir that leads to the formation of its active form, ganciclovir triphosphate, which terminates DNA synthesis through the competitive inhibition of CMV DNA polymerase (UL54 gene). Cidofovir and foscarnet are also used to treat CMV, with a mechanism of action on CMV DNA polymerase similar to that of HSV. Two additional more recently approved anti-CMV drugs are maribavir and letermovir. Maribavir inhibits the UL97 kinase, which is also responsible for a variety of other cellular functions that promote CMV survival. Letermovir inhibits the terminase complex (formed by UL56, UL89, and UL51 gene products), responsible for viral packaging (15).

Resistance to these drugs is mediated by mutations in the genes described above, as summarized in Table 1 (15). Ganciclovir resistance is most commonly caused by mutations in UL97, with the canonical mutations M460V, H520Q, C592G, A594V, L595S, and C603W accounting for approximately 80% of resistance (16). These mutations can confer varying degrees of resistance; patients with only low-grade resistance may respond to ganciclovir dose escalation in the absence of severe disease (29). UL54 DNA polymerase mutations are less common and usually develop on top of pre-existing UL97 mutations. Mutations in the UL54 exonuclease domain can confer ganciclovir–cidofovir cross-resistance, meaning that patients on prolonged ganciclovir therapy can become cidofovir-resistant, even if never clinically exposed. Mutations in other regions of UL54 can lead to foscarnet resistance and other cross-resistance combinations (15). Resistance to maribavir is most commonly mediated by the UL97 mutations T409M, H411Y, C480F, and F342Y, with great variability in the degree of resistance conferred. Low-grade maribavir resistance mediated by UL27 has been described predominantly in *in vitro* experiments and more recently for the first time clinically in a maribavir non-responder at baseline (17). *In vitro* selection experiments have demonstrated a low genetic barrier to letermovir resistance, mediated predominantly by UL56 and less commonly by UL89 and UL51 mutations. However, only UL56 mutations have been seen clinically, with mutations at the C325 locus conferring absolute resistance to letermovir being predominant (15, 18).

Even with the relative wealth of drug options, some patients will develop resistance to multiple drugs. Furthermore, each of these drugs is associated with some undesirable side effects, including marrow suppression (ganciclovir), nephrotoxicity (cidofovir), dysgeusia (maribavir), and gastrointestinal effects (maribavir and letermovir). Additionally, for maribavir and letermovir, variable insurance coverage and expensive copayments can lead to significant financial toxicity for some patients. Promising new anti-CMV therapies currently under development and investigation include filociclovir (cyclopropavir) and CMV-specific adoptive T-cell therapy (15, 30).

### Clinically available testing methodologies

As of 2024 in the United States, CMV drug resistance testing is available clinically using sequencing-based methods in several large reference labs and academic centers. Phenotypic testing is no longer used diagnostically due to challenges in standardization, long turnaround time, and labor-intensiveness. However, phenotypic testing, particularly recombinant phenotyping, continues to be instrumental in the research setting to confirm the validity of new drug resistance mutations identified by genotypic methods. The sequencing-based methods we use today are only possible thanks to decades of research rigorously genotypically and phenotypically characterizing each mutation that confers resistance and correlating these data with clinical trial data.

Clinical CMV drug resistance sequencing assays usually include at least limited regions of UL97 (for ganciclovir and maribavir resistance), UL54 (for ganciclovir, foscarnet, and cidofovir resistance), and/or UL56 (for letermovir resistance) (15, 39, 40). Some assays also include UL27 for maribavir resistance (39). Assays using either Sanger sequencing or NGS methodology are available—though an increasing number of reference laboratories have switched from Sanger to NGS methodologies in the past decade (39, 40). Practical reasons for this change include the decreasing cost of NGS, as well as the streamlined workflow of being able to amplify and sequence entire genes associated with resistance to all five drugs in a single reaction. Additionally, NGS can detect minor frequency variants with higher sensitivity, which may allow for the prediction of emerging drug resistance earlier than by Sanger sequencing methods. Although this potential advantage of NGS has been demonstrated in case reports, it is still unclear whether the identification of these low-frequency mutations at earlier time points is associated with better clinical outcomes (15, 41).

Published studies of both Sanger sequencing and NGS methods have reported heterogeneity in methods and the degree of quality control, as well as the lack of reproducibility of minor frequency or previously uncharacterized variants, especially in specimens with low viral load (18, 42). This may be due to sample misidentification, poor quality control, contamination, PCR artifact, sequencing artifact, or interpretation errors. When unusual or uncommon sequence variant results are obtained, laboratorians should consider a careful assessment of sequence quality and repeat testing from extraction; clinicians should consider reassessing whether the patient meets the criteria for treatment refractory CMV and repeating antiviral resistance testing on a new specimen (15). While drug resistance testing is typically performed on plasma, resistance mutations compartmentalized to the central nervous system (CNS) have been described; cerebrospinal fluid testing can be considered in patients with refractory CMV encephalitis (43).

### Clinical indications

CMV infection is a serious complication in transplant recipients and other immunocompromised patients. Although the drugs discussed above are effective for most patients in the prevention and treatment of CMV infection, they are each associated with important toxicities, and options are limited for those with resistance to multiple drugs. CMV drug resistance sequencing can help guide therapeutic management in the setting of refractory infection or disease.

Those at highest risk for the development of CMV drug resistance include those with prolonged antiviral drug exposure, high-risk stem cell transplant recipients (donor CMV IgG negative [D−]/recipient positive [R+] and D+/R+), and intestinal, multi-visceral, and lung transplant recipients (especially those D+/R−) (29). Resistance testing is indicated when an at-risk patient has refractory CMV infection or disease, as defined by a >1 log IU/mL increase in viral load or worsening signs, symptoms, or progression to end-organ disease, after at least 2 weeks of appropriately dosed antiviral therapy (29, 31). Resistance testing may also be considered when a patient has persistent infection or disease, as defined by a lack of response in viral load or signs and symptoms with appropriate therapy (29, 31). These definitions are established for trial protocols; although many use them as guidance in the clinical setting, some flexibility should be allowed for unique patient presentations. It is important to wait at least 2 weeks after starting therapeutic (not prophylactic) dosing of the drug to evaluate for treatment refractoriness, as it is common for viral loads to paradoxically increase or remain elevated in the first week after a drug is started. Lastly, though currently available methods have reported limits of detection ranging from 240 to 1,000 IU/mL, drug resistance testing on patients with viral loads <1,000 IU/mL should be considered with caution, as sequencing results at low viral loads may not be reproducible (29).

## HUMAN IMMUNODEFICIENCY VIRUS

### Drugs and mechanisms of resistance

The FDA has approved over 30 antiretroviral drugs belonging to eight mechanistic classes for the treatment of HIV since 1987 (32). This section focuses on HIV-1 and the most commonly used drug classes of INSTIs, NRTIs, NNRTIs, and PIs. Most patients with HIV in the United States are started on an initial regimen that includes a second-generation INSTI (i.e., bictegravir and dolutegravir) and one or two NRTIs (i.e., tenofovir, abacavir, emtricitabine, and lamivudine). Other initial treatment regimens may include the backbone therapy of two NRTIs accompanied by a first-generation INSTI (i.e., raltegravir and elvitegravir), protease inhibitor (i.e., darunavir and atazanavir), or NNRTI (i.e., doravirine, efavirenz, and rilpivirine) (32, 33). Resistance to all four of these drug classes is mediated by mutations in *pol*, a roughly 3 kb gene encoding a polyprotein that is processed into reverse transcriptase (target of NRTIs and NNRTIs), protease (target of PIs), and integrase (target of INSTIs).

NRTIs require phosphorylation by cellular kinases before becoming active to competitively inhibit nucleotide binding to reverse transcriptase and terminate the DNA chain. Resistance to NRTIs can be caused by mutations that decrease reverse transcriptase affinity for NRTIs over natural nucleotides (discrimination pathway) or by mutations that facilitate the removal of incorporated NRTI (excision pathway) (44). The most common mutation to develop in patients in whom first-line therapy fails is M184V/I, a discrimination pathway mutation that confers high-level resistance to lamivudine and emtricitabine and also reduces viral fitness and increases susceptibility to zidovudine and tenofovir (44). The most common excision pathway mutations are also referred to as thymidine analog mutations because they are selected by older thymidine analog drugs. Thymidine analog mutations are common in countries where thymidine analogs are still used. Although they are among the most common type of transmitted NRTI resistance mutation seen in patients in the United States, they have minimal effect on current first-line NRTIs (45).

NNRTIs do not require phosphorylation and non-competitively inhibit reverse transcriptase by binding to a hydrophobic pocket that causes conformational change of the active polymerase site. NNRTI resistance is mediated predominantly by mutations in amino acids lining this hydrophobic binding pocket region (44). The NNRTI resistance mutation K103N is the most common transmitted drug resistance mutation, with prevalence of 8.6% in the United States (45).

Protease is critical for Gag and Pol polyprotein processing into functional viral proteins. PIs are usually boosted with ritonavir or cobicistat for pharmacokinetic effect. Boosted PI regimens have high genetic barriers to resistance—virologic failure due to drug resistance is rare. When resistance does emerge, it is due to mutations in the substrate cleft or those that compensate for decreased fitness associated with substrate cleft mutations. The prevalence of transmitted PI resistance in the United States is 4.6% and is primarily mediated by mutations that have minimal effect on the preferred boosted darunavir regimens (32, 45).

After reverse transcription, integrase performs 3′ processing of HIV DNA and catalyzes strand transfer of the HIV DNA into host DNA, a critical step in HIV replication. INSTIs bind to the enzyme’s active site and block strand transfer, leading to the degradation of the HIV DNA complex. Mutations in and around this active site can confer variable resistance. Transmitted INSTI resistance in the United States is rare (<1%) and, thus, far stable, although a recent report from Italy described an increase in transmitted first-generation INSTI resistance mutations from 1.3% to 3.9% from 2014 to 2019 (46). Second-generation INSTIs have a high genetic barrier to resistance, and transmitted resistance is extremely rare although it has been reported (47). There is concern that as second-generation INSTIs continue to be used, including long-acting injectable formulations with a long elimination half-life (i.e., cabotegravir), resistance may increase.

In-depth explanations of the most common clinically significant mutations and mechanisms of resistance can be found at the Stanford University HIV Drug Resistance Database website (19, 48).

### Clinically available testing methodologies

The preferred HIV drug resistance testing method is genotypic on plasma HIV RNA, owing to the faster turnaround time and availability of an expertly curated drug resistance database used worldwide that standardizes interpretation (19, 32, 48). Conventional testing includes sequencing of the first 1.5 kb of the *pol* gene, to capture the protease and reverse transcriptase coding regions for the prediction of PI, NRTI, and NNRTI resistance. For patients who require INSTI resistance testing, the integrase coding region of the *pol* gene can be sequenced as well.

Both Sanger sequencing and NGS methodologies are now clinically available for plasma HIV RNA drug resistance testing. The Sentosa SQ HIV-1 Genotyping Assay (Vela Diagnostics, Fairfield, NJ), an NGS method that detects mutations associated with PI, NRTI, NNRTI, and INSTI resistance, received FDA approval in 2019. The assay uses an adapted Ion Torrent platform and automates most steps in the sequencing process from sample loading through data analysis, making HIV NGS drug resistance testing more accessible. However, reports have described assay or quality control failure rates ranging from 13% to 44% of runs, possible bioinformatic analysis errors, poor reproducibility of minority variant detection, and decreased ability to resolve low-frequency mutations compared to other NGS platforms (49–53).

Most published studies have demonstrated that, depending on the method and variant frequency cutoff, NGS methods will detect higher levels of resistance in 5%–20% more specimens than Sanger sequencing methods, owing to increased sensitivity for the detection of minority variants (variants that occur in <20% of sequences) (11, 50, 52, 54). The identification of minority variants is associated with an increased risk of virologic failure in patients on NNRTI-based therapy, but no such relationship has been established for NRTI-, PI-, or INSTI-based therapy (11, 55, 56). The importance of minority variants is likely inversely proportional to the strength of the genetic barrier of resistance to a given treatment regimen. Now that second-generation INSTI + NRTI regimens with high genetic barriers to resistance are predominantly used for first-line therapy, it is unclear whether the detection of minority variants is clinically useful. Indeed, reporting resistance when a patient is still likely to respond to a given drug could be harmful. A 2018 study reported that although rates of virologic failure were higher for individuals with drug-relevant minority variants, all 27 study participants who experienced virologic failure reported poor drug adherence. Of these 27 individuals, all 19 who subsequently improved their treatment adherence later experienced viral suppression without their clinical provider knowing that minority variants were present (11). Medication adherence is likely a confounding factor that must be accounted for when considering the relationship between minority variants and virologic failure.

Other less commonly used HIV drug resistance testing methods include proviral HIV DNA sequencing from peripheral blood mononuclear cells, phenotypic recombinant viral assays, and testing of specimen types other than blood. Proviral HIV DNA sequencing may detect mutations undetected by conventional plasma RNA testing, providing information about the latent pool of resistance-associated mutations that could re-emerge under selective drugs (57). However, the method also can miss mutations identified by RNA testing due to viral kinetics. There are also limitations in specificity related to the detection of APOBEC-mediated mutations (58). Phenotypic recombinant viral assays can help in assessing the impact of uncommon mutation combinations and interactions but are labor-intensive and have slow turnaround times compared to genotypic methods. Proviral sequencing and phenotypic resistance testing data should always be interpreted in the context of all prior available plasma HIV RNA drug resistance sequencing data (32). Rarely, patients may present with virologic failure compartmentalized to the CNS despite relative plasma suppression; resistance testing from the CSF can be considered for these cases (32, 59).

### Clinical indications

Clinical indications for HIV genotypic drug resistance testing are broad because of the prevalence of transmitted drug resistance in treatment-naïve individuals and the lifelong nature of treatment. Testing for NRTI, NNRTI, and PI resistance is indicated in the following settings: (i) acute HIV infection, (ii) entry into care regardless of timing for starting therapy, (iii) virologic failure with HIV RNA viral load >1,000 copies/mL (can consider for viral loads 200–1,000 copies/mL although the likelihood of success may be lower), (iv) suboptimal HIV RNA suppression (suppression not achieved within 24 weeks on therapy), and (v) pregnancy (32, 33). Testing for INSTI resistance is indicated in the setting of prior long-acting cabotegravir pre-exposure prophylaxis, virologic failure on an INSTI-based regiment, or suspected transmission from an individual with INSTI resistance (32, 33). Practically, with NGS-based methods, NRTI, NNRTI, PI, and INSTI resistance interpretations may be provided in a single test.

For those with acute HIV infection or entering into care, treatment should not be delayed while waiting for resistance testing results. For those with virologic failure or suboptimal suppression, it is recommended to conduct resistance testing while the patient is still on the failed regimen or within 4 weeks of treatment discontinuation. Selection of drug regimen in the setting of virologic failure should take into account all available historical resistance testing data (32, 33).

There are rare scenarios where resistance testing data are needed for clinical decision-making, but plasma HIV RNA sequencing may be unsuccessful or insufficient. Proviral HIV DNA sequencing can be considered in patients with virologic suppression on a stable drug regimen, those with persistent low-level viremia, those with virologic failure who recently discontinued therapy, and those potentially infected with a drug-resistant virus (32, 57, 58). Phenotypic recombinant viral assays can be considered for patients who have experienced multiple regimen failures and have a large number of historical resistance mutations (32).

## UNCOMMONLY USED CLINICAL ANTIVIRAL RESISTANCE TESTS

As new drugs come to market and viruses evolve, indications and methods for antiviral resistance testing can change rapidly. Antiviral resistance testing continues to be available for limited indications for influenza, hepatitis B, and hepatitis C viruses, which are summarized below.

For influenza viruses, neuraminidase inhibitors (oseltamivir, zanamivir, and peramivir) have been the mainstay of treatment and chemoprophylaxis since the emergence of widespread adamantane resistance in the mid-2000s. Since the introduction of the H1N1pdm09 strain of influenza A, transmitted neuraminidase resistance has been rare, though occasional cases and clusters have been reported (20). According to the CDC’s weekly US influenza surveillance report, less than 0.3% of surveilled influenza viruses have been resistant to neuraminidase inhibitors thus far in the 2023–2024 influenza season. The new viral polymerase acidic (PA) cap-dependent endonuclease inhibitor baloxavir was approved in 2018; reduced susceptibility to baloxavir has been reported on treatment in up to 20% of individuals, although transmitted resistance is rare (10, 60). Influenza drug resistance testing (genotypic with phenotypic confirmation) is available in US public health labs and should be considered in influenza-infected patients whose infection has not responded to treatment, especially those who are immunocompromised (8, 34, 61). A summary of testing methods and amino acid substitutions associated with influenza resistance is available through the World Health Organization (8).

Development of HBV resistance is extremely rare in patients on nucleoside/tide analogs with a high genetic barrier to resistance such as entecavir or tenofovir, which are now recommended as first-line therapies. Transmitted resistance is rare in the United States (1.2%) as assessed by Sanger sequencing methods (21). Drug resistance in treatment-naive patients was approximately 1% over 5 years on entecavir monotherapy and was 0% on tenofovir disoproxil fumarate treatment for up to 8 years (35). As such, HBV drug resistance testing should be considered mainly in the setting of treatment failure, especially among individuals who may have previously received drugs like lamivudine with a lower genetic barrier to resistance, which could confer cross-resistance to entecavir (35). Clinical resistance testing is available in reference labs by lab-developed sequencing-based methods targeting known mutations in the reverse transcriptase coding region of the *pol* gene (21, 22). Both publicly available and commercial databases/software exist for interpretation including DeepChek-HBV/SeqHepB (Advanced Biological Laboratories), HBVrtDB, and HBVdb (23, 62).

Direct-acting antivirals (DAAs) have entirely revolutionized HCV treatment in the past decade, with drug combinations of sofosbuvir/velpatasvir and glecaprevir/pibrentasvir being recommended as first-line therapy regardless of genotype (36). Resistance-associated substitutions (RASs) in the genes encoding NS5A (replication complex), NS3 (protease), and NS5B (polymerase) have been variably associated with failure to achieve sustained virologic response (SVR) on therapy. Of note, while some NS5A RASs are currently clinically relevant, NS3 and NS5B RASs generally are not (24, 36). NS3 RASs are frequently detected at baseline and after virologic failure but have not been associated with decreased SVR to currently recommended DAA combinations in the United States. NS5B inhibitors have a high genetic barrier to resistance; RASs are rare and have not been associated with decreased SVR, likely due to low relative fitness (24). NS5A drug resistance sequencing for specific RASs should be considered for specific situations where combinations of DAA regimen and patient characteristics would make this data clinically meaningful (24, 25, 36). Though there are no FDA-approved assays, this testing is available in a handful of reference labs as lab-developed tests. Both publicly and commercially available databases are available for interpretation, including via SmartGene (SmartGene Inc.), DeepChek-HCV Software (Advanced Biological Laboratories), and HCV-GLUE (25, 63).

## FUTURE DIRECTIONS

In the coming years, clinical antiviral resistance testing is likely to increasingly rely on genotypic rather than phenotypic methods and to move from Sanger sequencing to NGS methods. This change will largely be due to logistic and financial pressures, medical laboratory scientist labor shortages, and decreased education and training in viral culture techniques. As these transitions happen, it will be important for research studies and drug trials to correlate resistance data obtained via NGS methods to clinical response and outcomes data. The capability to conduct phenotypic testing should be maintained in select research, industry, and public health laboratories to aid in drug discovery, characterization of new mutations, and clinical trials.

The availability of clinical antiviral drug resistance assays in the coming years will also depend on the outcomes of ongoing efforts by the FDA to increase oversight of lab-developed testing (64). Increased regulatory oversight may help to standardize some tests, particularly those that are frequently used. However, drug resistance testing for most viruses (currently conducted exclusively as lab-developed testing) is likely to become less available, or potentially unavailable, given limited financial incentives for device manufacturers.

In the future, we hope to see clinical availability of antiviral resistance testing for small-molecule drugs and monoclonal antibody therapies for viruses such as SARS-CoV-2, respiratory syncytial virus, mpox, and VZV (65–68). As NGS methods mature, viral sequencing assays employing techniques such as multi-virus primer pools, hybrid capture, or metagenomics may allow for more flexible protocols and streamlined workflows (49). Artificial intelligence might be harnessed to model and predict antiviral resistance, in combination with currently existing rule-based algorithms (69). On the opposite end of the spectrum, advancements in microfluidics, microelectronics, and molecular biology are making highly multiplexed nucleic acid amplification testing more accessible, which could allow for the assessment of the most common or important resistance mutations at the point of care (70).

## CONCLUSIONS

Over the past decade, clinical antiviral resistance testing has moved from phenotypic to genotypic methods. Increasingly, genotypic methods are first-line, with phenotypic methods used only in complex or investigational cases. Correlation between phenotypic, genotypic, and clinical outcomes data is key for establishing new resistance testing methods. Drug resistance testing by NGS is still an emerging methodology—more standardization and clinical outcomes correlation are needed, especially for establishing the significance of minority variant detection. The landscape of clinical antiviral resistance testing will continue to change quickly due to emergent technology, the rapidity of viral evolution, and the accelerated pace of drug development.
